# The antiseizure effect of resveratrol on pentylenetetrazole-induced seizures during different phases of the estrous cycle in rats

**DOI:** 10.1016/j.ibneur.2026.10.010

**Published:** 2026-10-24

**Authors:** Shahin Hassanpour, Seyed Amirreza Dehghani, Kiana Rastegar, Seyed Mehdi Sarmanrei

**Affiliations:** Department of Veterinary Basic Sciences, SR.C., Islamic Azad University, Tehran, Iran

**Keywords:** Antiepileptic, Antiseizure, Resveratrol, Estrous cycle

## Abstract

This research investigated the effects of resveratrol on seizure suppression in a rat model of catamenial epilepsy, focusing on variations through the estrus cycle. Female Wistar rats were randomly divided into five groups: control (saline), valproic acid (VPA, 75 mg/kg), and resveratrol at doses of 20, 40, and 80 mg/kg. Each group was further divided into four subgroups corresponding to the phases of the estrus cycle (proestrus, estrus, metestrus, and diestrus). Rats received intraperitoneal injections of either saline or pentylenetetrazol (PTZ, 80 mg/kg). Seizure initiation times and durations for myoclonic (ITMS) and tonic-clonic seizures (ITTS) were recorded. Blood samples were collected to assess serum concentrations of MDA, SOD, GPx, TNF-α, and IL-1β. Resveratrol at 80 mg/kg significantly increased both ITMS and ITTS throughout all estrous phases compared to controls (p ˂0.05). Both VPA and resveratrol treatment significantly reduced seizure duration (p ˂0.05). Resveratrol demonstrated a more pronounced antiseizure effect during the metestrus and diestrus phases. Resveratrol decreased MDA levels and increased SOD and GPx activity (p ˂0.05). TNF-α and IL-1β were also reduced following resveratrol administration (p ˂0.05). Overall, resveratrol decreased seizures in PTZ-treated rats, with it being more effective during the luteal phase compared to the follicular phase.

## Introduction

Epilepsy is among the most prevalent neuro-cerebral disorders in humans and is characterized by recurrent seizures due to neurological dysfunction. A seizure is defined as abnormal synchronized neuronal activity in the brain, which causes transient symptoms such as sensory disturbances, involuntary muscle contractions, amnesia, and loss of consciousness (Falco-Walter, 2020). Catamenial epilepsy is a form of epilepsy where seizures are exacerbated during specific phases of women’s menstrual cycle (Reddy, 2016). Fluctuations in the ovarian hormones during the estrous cycle and electrolyte imbalance are considered the main causative factors of catamenial epilepsy. Progestogen has anticonvulsant effects while estradiol exhibits proconvulsant properties, indicating that steroid hormones can influence both the initiation and spread of seizures. In rodents, a correlation was found between serum estradiol/progesterone levels and the frequency of seizures during ovulatory cycles (Ajayi and Akhigbe, 2020, Reddy, 2016). Although several mechanisms have been implicated, variations in ovarian hormone secretion are believed to play a significant role in catamenial epilepsy; the estrogens are widely recognized to be proconvulsant, while progesterone has pronounced anticonvulsant effects (Li et al., 2024).

As the brain’s main inhibitory neurotransmitter, gamma-aminobutyric acid (GABA) plays a central role in the pathogenesis of epilepsy, with reduced GABA activity or impaired GABA signaling predisposing neurons to uncontrolled excitation and seizures (Perucca et al., 2023). Although the pathogenesis of catamenial epilepsy is not fully understood, GABA channels, which exhibit sedative and anti-anxiety properties, are thought to play a pivotal role. Progesterone and its metabolites allopregnanolone and pregnanolone impact GABAergic receptors and reduce the occurrence of seizures (Stefaniak et al., 2023). In contrast, estrogen reduces GABAergic inhibition by reducing GABA receptor function or expression, leading to increased neuron excitability and risk of seizure (Joshi and Kapur, 2019). PTZ, which is frequently utilized to induce generalized seizures in experimental epilepsy, functions by inhibiting GABA_A_ receptors (Zhang et al., 2024).

Common antiseizure medications that modulate enzymes, like carbamazepine, phenobarbital, and sodium valproate, also affect the serum concentrations of steroid hormones (Dalkilic, 2021). Thus, there is an increasing interest in strategies for treating epilepsy which do not affect steroid levels. Medicinal plants and plant derivatives have attracted attention for this aim (Zhang et al., 2024). Resveratrol is a natural polyphenol found in red wine, grapes, berries, peanuts, and dark chocolate, and has multiple biological functions that may be beneficial in metabolic diseases (Vikal et al., 2024). It is known for its antioxidant and anti-inflammatory properties and improves mitochondrial function, lipid oxidation, and insulin sensitivity through activation of sirtuin 1 and adenosine monophosphate-activated protein kinase (Najafi et al., 2021). Resveratrol mitigates oxidative stress and reduces proinflammatory cytokines (Fraiz et al., 2021). Resveratrol was reported to have a protective effect on the brain in kainic acid-induced rat epilepsy model (Li et al., 2017). Treatment with resveratrol reduced the kainic acid-induced rise in the glutamate/GABA ratio in the hippocampus. The antiepileptic properties of resveratrol could be partly due to lowering glutamate-induced excitotoxicity and increasing GABAergic inhibition (Li et al., 2017).

The interplay of hormones and epilepsy is complex and has diverse manifestations. Clinically, the effect of hormones on brain excitability manifests as variations in seizure frequency with changing hormone levels. This is most commonly observed in catamenial epilepsy (Li et al., 2024). Neurosteroids are recognized for their acute non-genomic effects through the direct modulation of NMDA and GABAA receptors, and primarily regulate neuronal excitability by interacting with synaptic and extra-synaptic receptors (Cutler et al., 2023). Resveratrol positively influences estrogen metabolism and sex steroid hormone binding globulin in females (Briansó-Llort et al., 2022). According to the literature, there is a link between resveratrol and sex hormones. We hypothesize that resveratrol can produce an anti-seizure effect on pentylenetetrazol (PTZ)- induced seizures. Thus, this study is aimed at assessing the antiseizure effect of resveratrol on PTZ-induced seizures at various stages of the estrous cycle in rats.

## Material and methods

### Animals

A total number of 200 female Wistar rats (8 weeks old, average weight: 200 g) were checked for vaginal smears to verify sexual puberty, and after the occurrence of two successive regular estrous cycles rats were randomly divided into 5 experimental groups (n = 40 per group). Each group investigated during 4 estrous cycle phases (proestrus, estrus, metestrus, diestrus) which 40 rats in each group randomly divided into estrous cycle phases (n = 10 per each phase). In the study, standard lab conditions were maintained with a temperature of 22 ± 1 °C and a 12-hour dark/light cycle, and the test subjects were given unlimited access to food and water (Anthony et al., 2026). Moreover, synchronization of estrous phases was conducted to coordinate the phases among the rats (Mattson and Cramer, 1985). All experimental procedures were approved by research committee of the Islamic Azad University, Science and Research Branch, Tehran. Iran (SRBIAU. 1404.3021).

### Synchronization of the estrous cycle

Before the commencement of the investigation, the evaluation of sexual puberty was validated by employing vaginal smears. The selection of the rats was based on the utilization of two regular estrous cycles (Mattson and Cramer, 1985). Prior to conducting the experiments, vaginal smears were collected each day to identify the phase of the estrous cycle according to the predominant cell type. Large round nucleated cells suggested proestrus, clusters of cornfield squamous epithelial cells indicated estrous, round nucleated epithelial cells with leukocyte infiltration represented metestrus, and the prevalence of leukocytes was identified as diestrus (Mattson and Cramer, 1985). Prior to the start of the study, the stage of the estrous cycle was assessed through daily vaginal smears and identification of the predominant cell types, including large round nucleated cells (proestrus), aggregates of cornified squamous epithelial cells (estrus), round nucleated epithelial cells with leukocyte infiltration (metestrus), and dominance of leukocytes (diestrus) (Dzirbíková et al., 2022). Vaginal smears was don at same time every day and checked by microscope. Moreover, the serum concentrations of estradiol and progesterone were assessed (Kaboutari et al., 2012).

### Drug preparation and administration

In this procedure, five groups were established: control (saline), valproic acid (VPA, 75 mg/kg), resveratrol (20 mg/kg, i.p), resveratrol (40 mg/kg, i.p), and resveratrol (80 mg/kg, i.p) (Table 1). PTZ was injected 30 min after the first injection. The subjects were selected at random, with each group made up of 10 adult female rats. Each group included animals from all four phases of the estrous cycle. In each group, rats received saline, VPA or resveratrol followed by PTZ administration. Each group was given saline or PTZ (80 mg/kg) via intraperitoneal injection. Resveratrol and PTZ was dissolved in saline and all injection volumes (for VPA, PTZ and resveratrol) were 0.5 mL. The dosage for PTZ (Kaboutari et al., 2012), resveratrol (Zarebavani et al., 2023) and VPA (Zahedi et al., 2024) was obtained for pilot study and previous reports.

**Table 1:** Treatment procedure.

**Group estrous cycle**	**First injection**	Second injection*
**Control**	Normal saline	PTZ (80 mg/kg)
Valproic acid	Valproic acid (75 mg/ kg)	PTZ (80 mg/kg)
Resveratrol (20 mg/kg)	Resveratrol (20 mg/kg)	PTZ (80 mg/kg)
Resveratrol (40 mg/kg)	Resveratrol (40 mg/kg)	PTZ (80 mg/kg)
Resveratrol (80 mg/kg)	Resveratrol (80 mg/kg)	PTZ (80 mg/kg)

Pentylentetrazole (PTZ) * 30 min after the first injection

### Study procedure

Seizures were induced and the subjects' behavior was observed for the subsequent 30 min to measure the initiation time of myoclonic seizures (ITMS), the initiation time of tonic–clonic seizures (ITTS), and seizure duration (SD) (Meritam Larsen et al., 2023). Seizures were graded according to a 5-stage system as follows: stage 0 - no behavioral alterations; stage I - twitching and spasms in facial and ear muscles; stage II - myoclonic contractions without an upright position; stage III - myoclonic contractions and standing posture with bilateral clonic movements of the forelimbs; stage IV - tonic-clonic seizures; stage V - generalized tonic-clonic seizures accompanied by postural instability. All protocol was done two blind method using sole operator. To minimize the influence of circadian rhythms on seizure susceptibility, all experiments were conducted between 9 a.m and 12 p.m (Kaboutari et al., 2012).

### Biochemical analysis

Immediately after ITMS, ITTS and SD, animals were euthanized via an intraperitoneal overdose (50 mg/kg) of sodium thiopental (Rotexmedica, Germany; following AVMA Guidelines for the Euthanasia of Animals 'No: S5.2.1.1′, Acceptable Methods; non-inhaled agents). Blood samples were taken from the cardiac of the subjects, centrifuged and serum malondialdehyde (MDA Catalog No. ZB-MDA-48A), superoxide dismutase (SOD Catalog No. ZB-SOD-48A), glutathione peroxidase (GPx Catalog No. ZB-GPX- A48), tumor necrosis factor alpha (TNF-α Catalog No. ZB-10764C-R9648) and interleukin-1 beta (IL-1β Catalog No. ZB-1510–01B-10) levels were determined using the corresponding assay kits (ZellBio GmbH).

### Statistical analysis

Data analyses were performed with SPSS by one-way analysis of variance (ANOVA) along with Tukey–Kramer multiple comparison post hoc tests. The data are expressed as mean ± SD (p < 0.05).

## Results

The mean serum levels for the estradiol and progesterone in proestrous, estrous, metsterous and diestrous were (83.1 ± 3.21 pg/mL, 38.2 ng/mL), (44.2 ± 1.89 pg/mL, 74.67 ± 3.36 ng/mL), (32.45 ± 1.65 pg/mL, 36.22 ± 1.15 ng/mL) and (33.23 ± 1.39 pg/mL, 26.37 ± 1.13 ng/mL), respectively.

Fig. 1 illustrates the effect of resveratrol on the ITMS at various phases of the estrous cycle. The administration of VPA (75 mg/kg) caused a significant increase in the ITMS throughout all phases when compared to the control group. Similarly, administering resveratrol (80 mg/kg) notably extended the ITMS in all phases relative to controls (p < 0.05). Additionally, a notable difference in ITMS was noted between the follicular (proestrus and estrus) and luteal (metestrus and diestrus) phases. Resveratrol (80 mg/kg) achieved a more significant extension of ITMS in the luteal phase (metestrus and diestrus) compared to the follicular phase (proestrus and estrus) (p < 0.001).

**Fig. 1:**
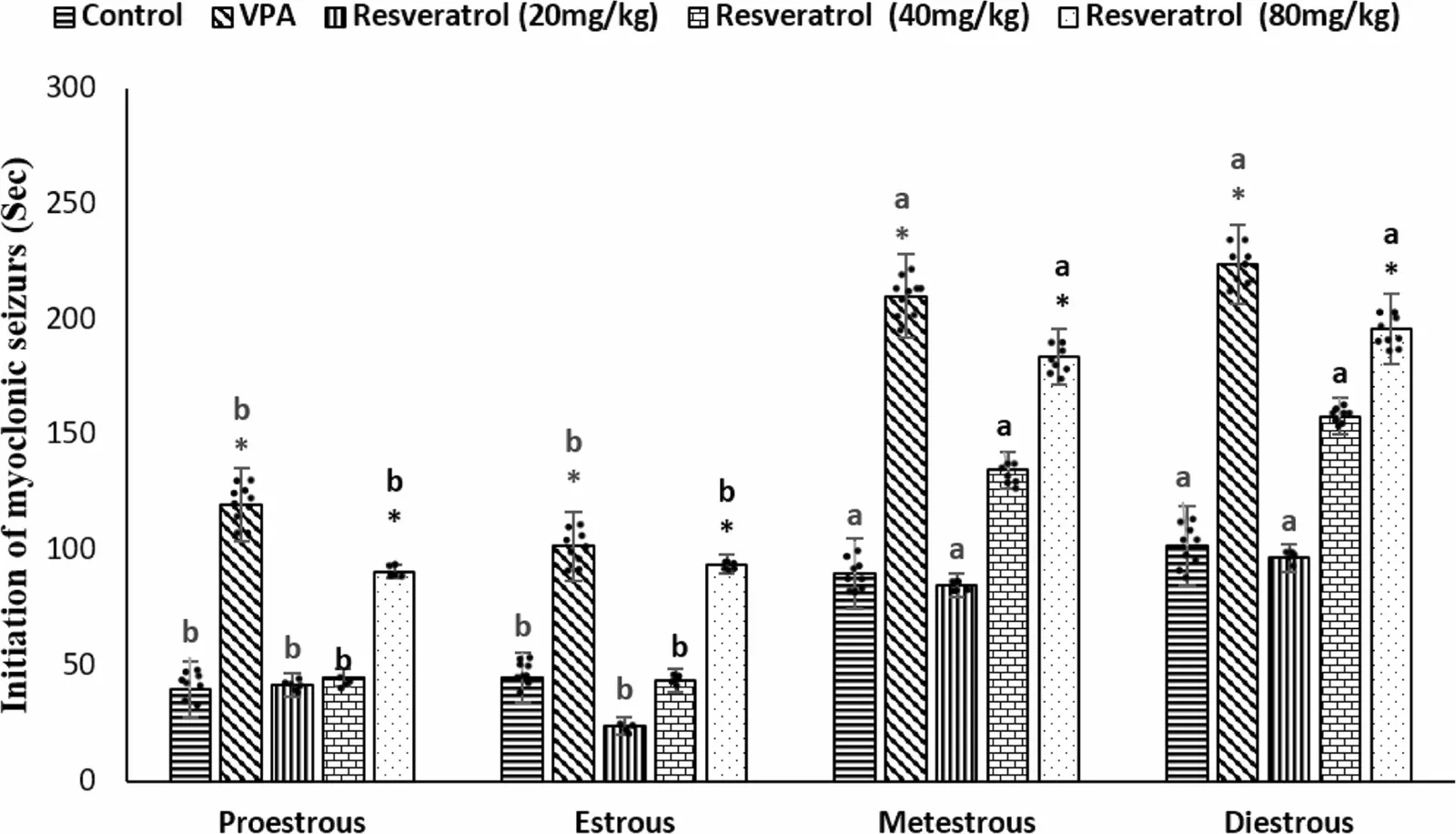
Effect of valproic acid (VPA) and resveratrol on the latency to onset of myoclonic seizures (ITMS) (s) across different stages of the estrous cycle. * In each phase of the estrous cycle, asterisks represent statistically notable differences in comparison with the control group (P < 0.05). For each group in every phase of the estrous cycle, distinct letters (a and b) represent significant differences (P < 0.05). Data are presented as mean ± SD.

As shown in Fig. 2, the administration of VPA (75 mg/kg) resulted in a notable increase in ITTS during each estrous phase compared to the control group (p < 0.05). Throughout all phases of estrus, resveratrol (80 mg/kg) notably extended ITTS when compared to the control group (p < 0.05). A notable difference in ITTS was also recorded between the follicular and luteal phases. resveratrol (80 mg/kg) noticeably extended the ITTS during the metestrus and diestrus phases in comparison to proestrus and estrus (p < 0.001).

**Fig. 2:**
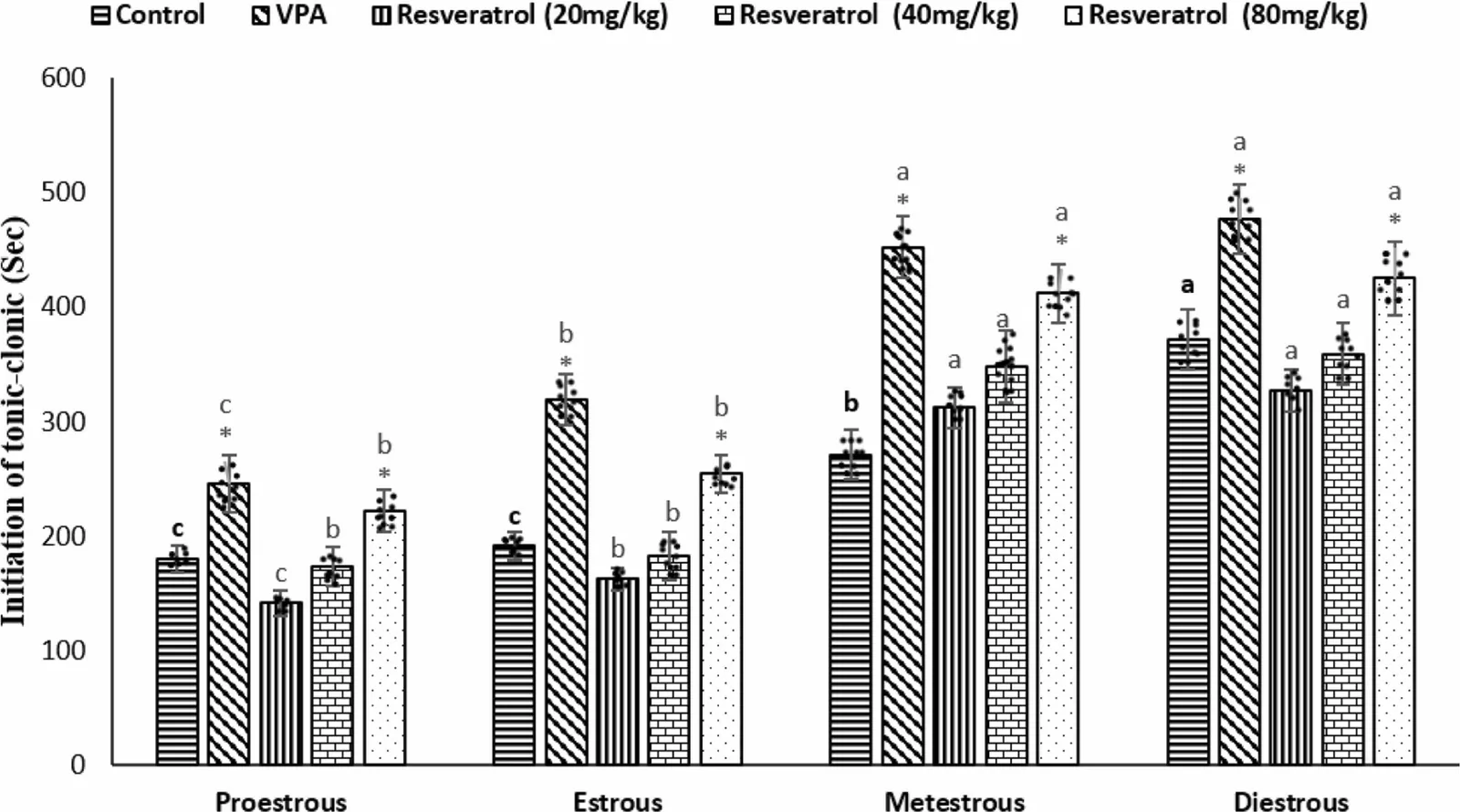
Effect of valproic acid (VPA) and resveratrol on the latency to onset of tonic–clonic seizures (ITTS) (s) across different stages of the estrous cycle. * In each phase of the estrous cycle, asterisks represent statistically notable differences in comparison with the control group (P < 0.05). For each group in every phase of the estrous cycle, distinct letters (a and b) represent significant differences (P < 0.05). Data are presented as mean ± SD.

VPA (75 mg/kg) notably reduced SD during all estrous stages in comparison to the control group, as shown in Fig. 3(p < 0.05). Compared to control, resveratrol (80 mg/kg) also markedly reduced SD across all phases of the estrous cycle (p < 0.05). A notable difference in SD was also detected between the follicular and luteal phases. Resveratrol (80 mg/kg) notably decreased SD in the follicular phase when compared to the luteal phase (p < 0.001).

**Fig. 3:**
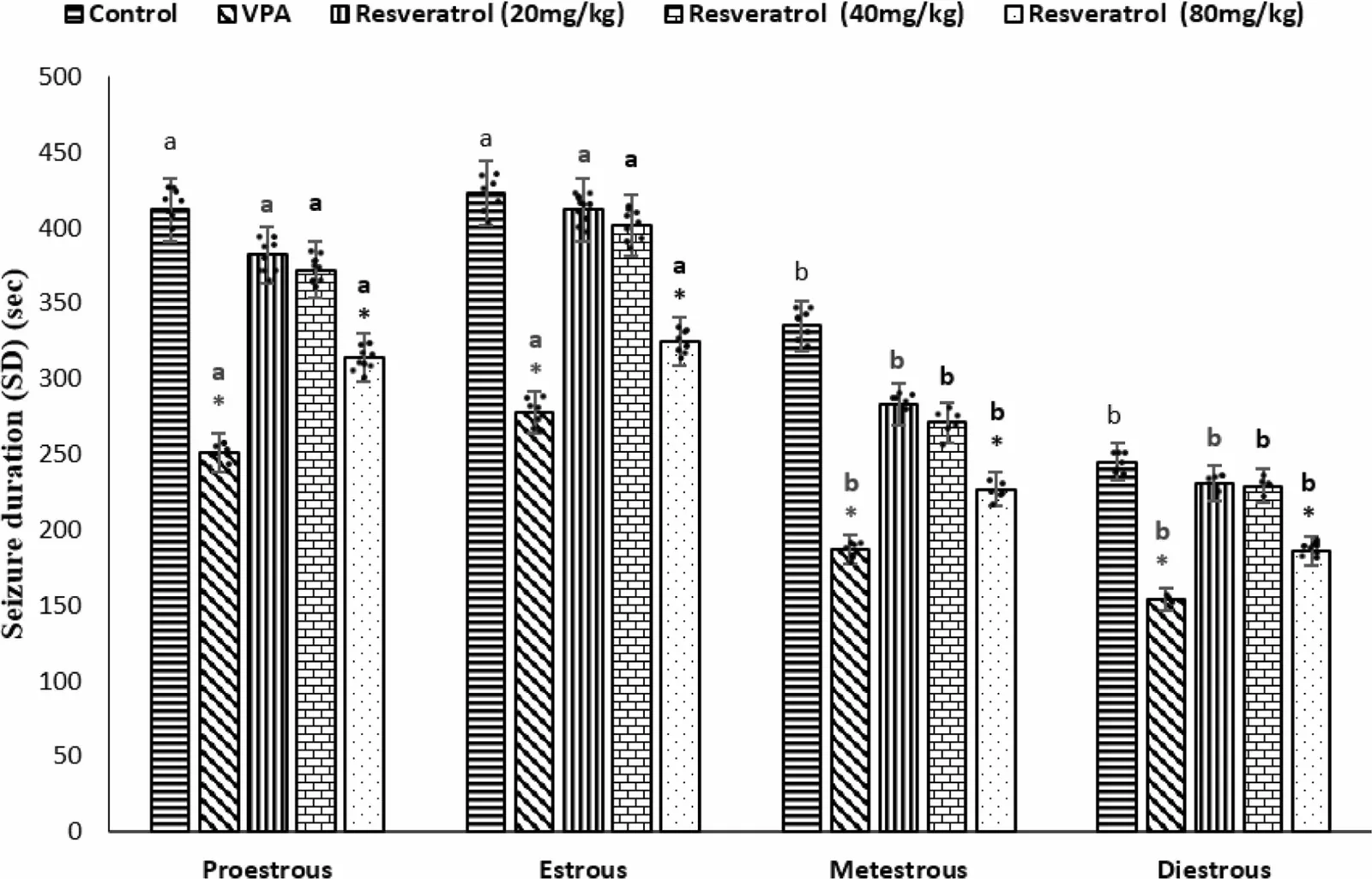
Effect of valproic acid (VPA) and resveratrol on seizure duration (s) across different stages of the estrous cycle. * In each phase of the estrous cycle, asterisks represent statistically notable differences in comparison with the control group (P < 0.05). For each group in every phase of the estrous cycle, distinct letters (a and b) represent significant differences (P < 0.05). Data are presented as mean ± SD.

As illustrated in Fig. 4. VPA did not significantly influence serum MDA, levels when compared to the control group (p > 0.05), while resveratrol (40 and 80 mg/kg) notably reduced serum MDA [F 4, 40 = 2.87] compared to the control group (p < 0.001).

**Fig. 4:**
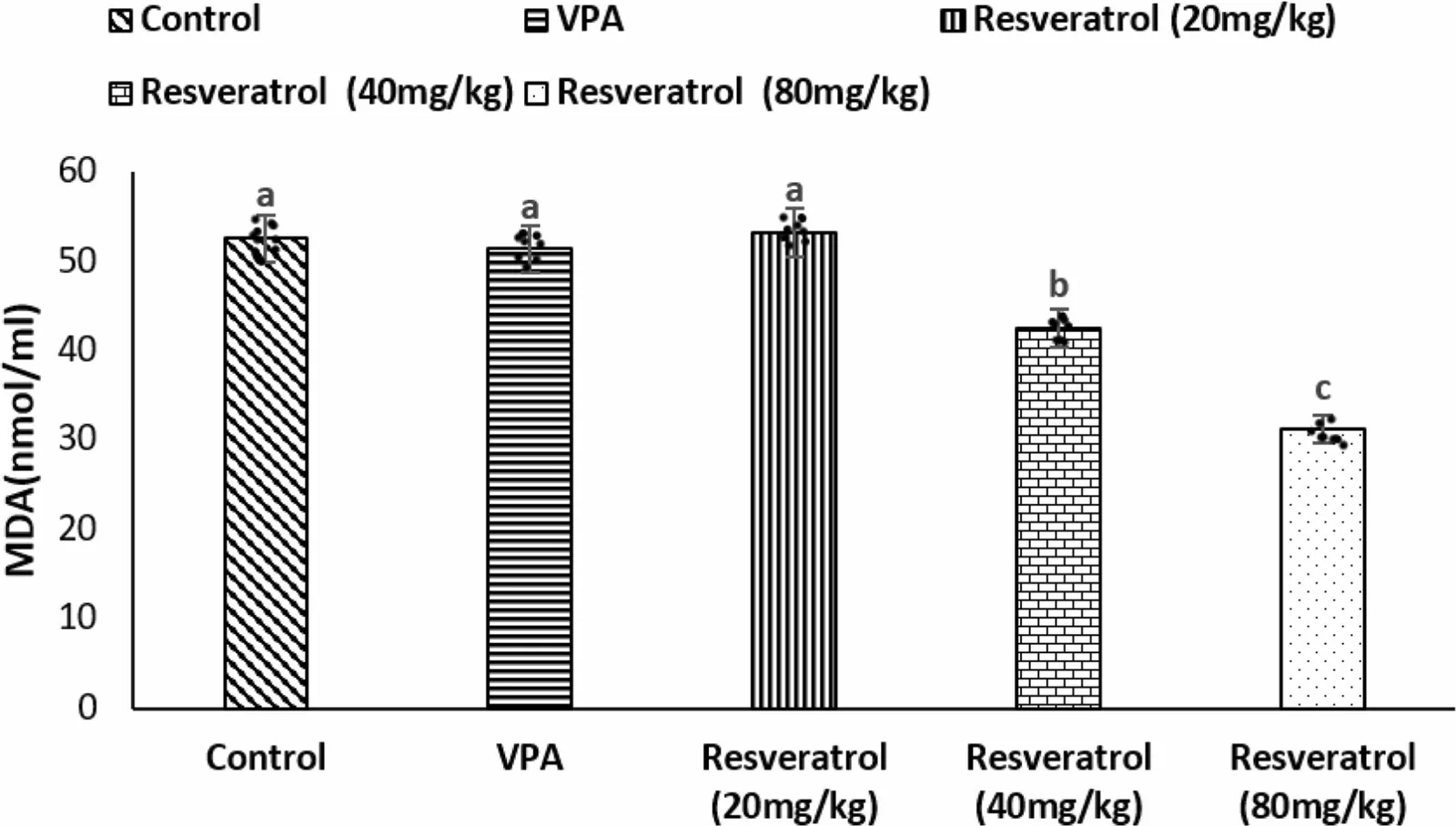
effects of the resveratrol (20, 40, and 80 mg/kg) on serum malondialdehyde (MDA) levels during PTZ-induced seizure in rat. For each group, different letters (a, b and c) represent significant differences (P < 0.05). Data are presented as mean ± SD.

As presented in Fig. 5, VPA did not significantly influence serum SOD levels when compared to the control group (p > 0.05), while resveratrol (40 and 80 mg/kg) notably increased serum SOD [F 4, 40 = 5.32] compared to the control group (p < 0.001).

**Fig. 5:**
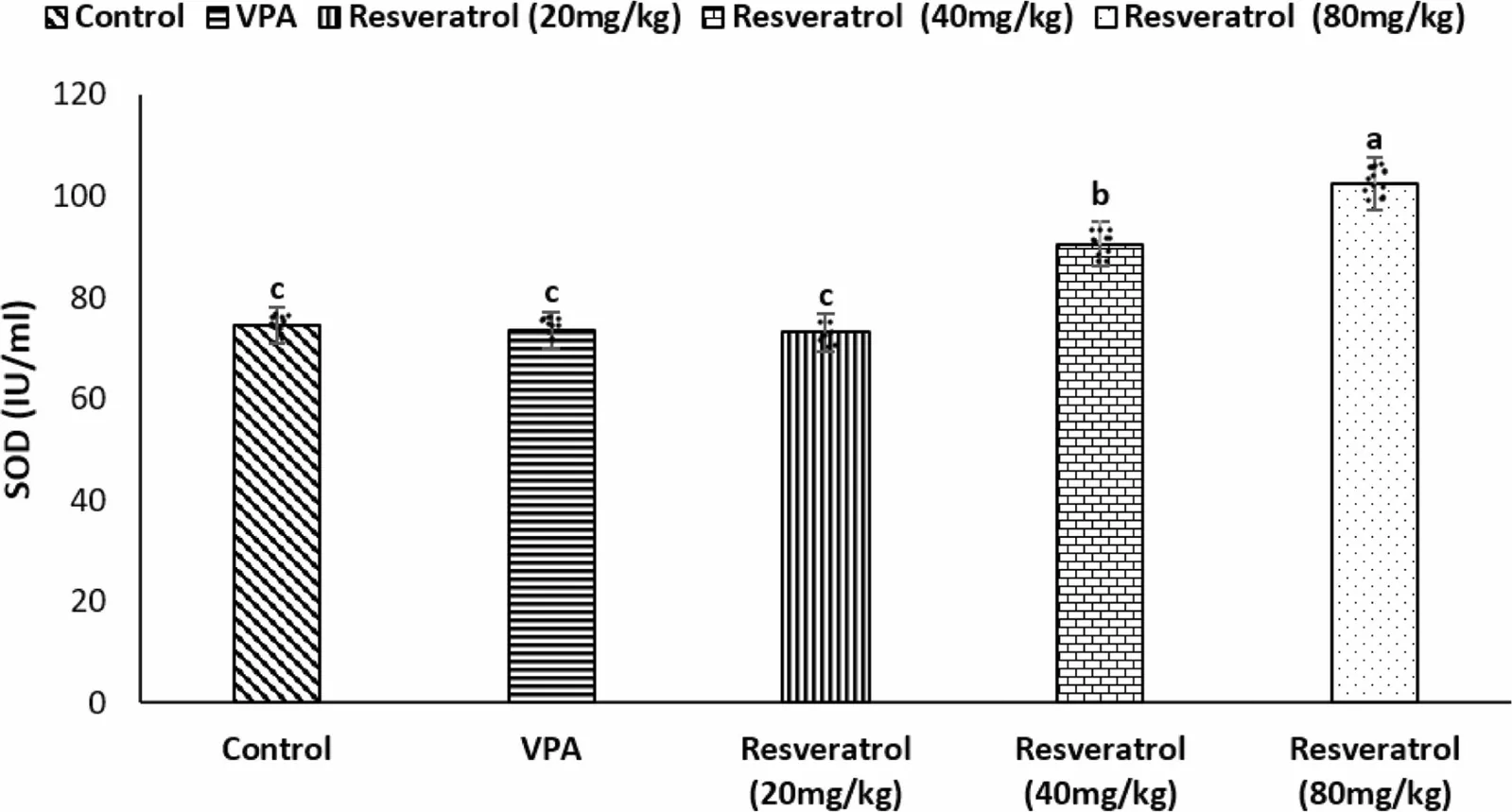
effects of the resveratrol (20, 40, and 80 mg/kg) on serum superoxide dismutase (SOD) levels during PTZ-induced seizure in rat. For each group, different letters (a, b and c) represent significant differences (P < 0.05). Data are presented as mean ± SD.

As shown in Fig. 6, VPA did not significantly influence serum GPx levels when compared to the control group (p > 0.05), while resveratrol (40 and 80 mg/kg) notably increased serum GPx levels [F 4, 40 = 5.72] compared to the control group (p < 0.001).

**Fig. 6:**
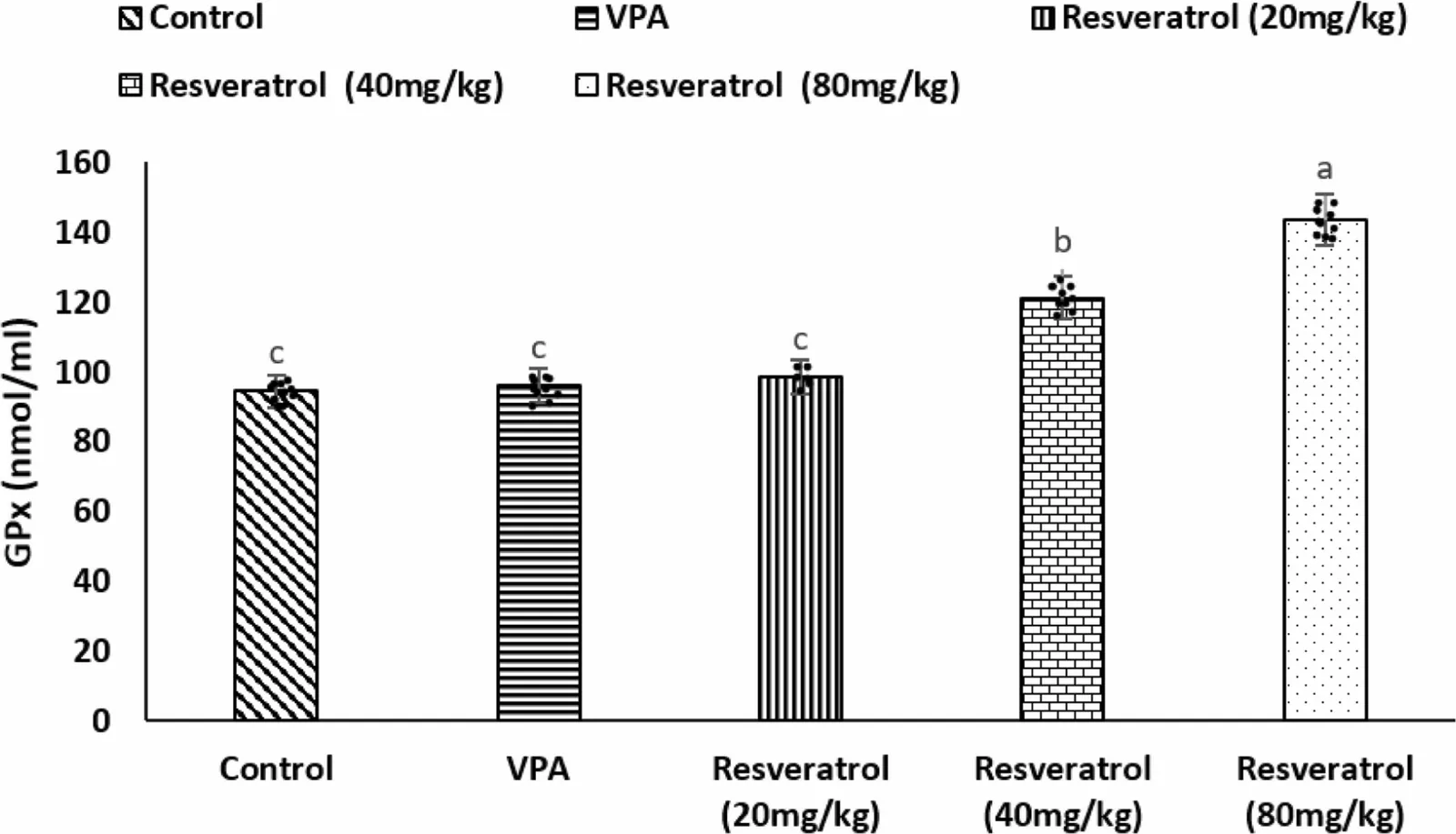
effects of the resveratrol (20, 40, and 80 mg/kg) on serum glutathione peroxidase (GPx) levels during PTZ-induced seizure in rat. For each group, different letters (a, b and c) represent significant differences (P < 0.05). Data are presented as mean ± SD.

Based on Fig. 7, VPA did not significantly influence serum TNF-α levels when compared to the control group (p > 0.05), while resveratrol (40 and 80 mg/kg) notably reduced serum TNF-α [F 4, 40 = 3.11] levels compared to the control group (p < 0.001).

**Fig. 7:**
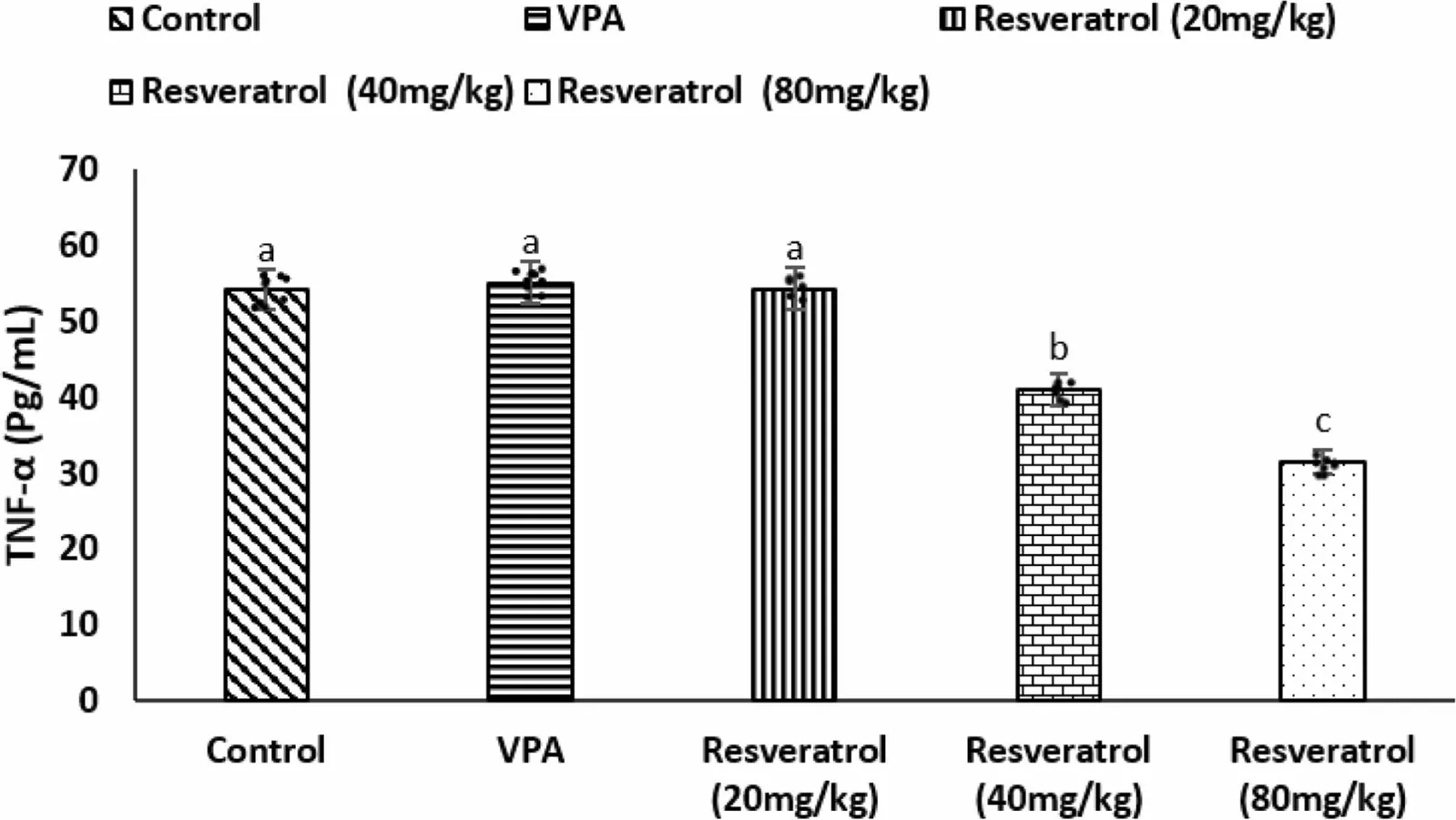
effects of the resveratrol (20, 40, and 80 mg/kg) on serum TNF-α levels during PTZ-induced seizure in rat. For each group, different letters (a, b and c) represent significant differences (P < 0.05). Data are presented as mean ± SD.

As seen in Fig. 8, VPA did not significantly influence serum −1β and TNF-α levels when compared to the control group (p > 0.05), while resveratrol (40 and 80 mg/kg) notably reduced serum IL-1β levels [F 4, 40 = 3.54] compared to the control group (p < 0.001).

**Fig. 8:**
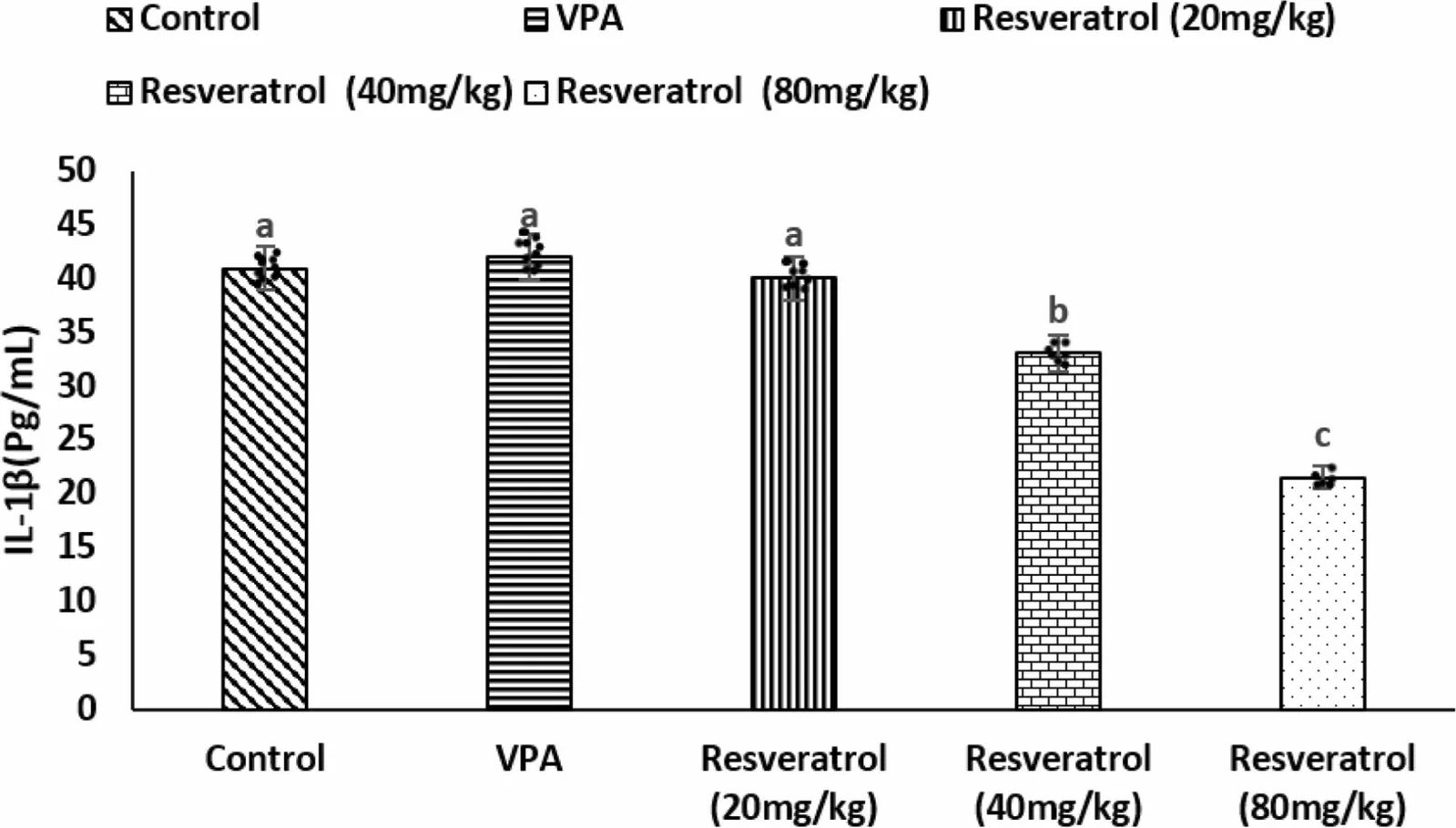
effects of the resveratrol (20, 40, and 80 mg/kg) on serum IL-1β levels during PTZ-induced seizure in rat. For each group, different letters (a, b and c) represent significant differences (P < 0.05). Data are presented as mean ± SD.

## Discussion

Based on the findings, resveratrol notably enhances both ITMS and ITTS throughout all stages of the estrous cycle. The duration of seizures was notably decreased in both the VPA and resveratrol treatment groups. Resveratrol demonstrated a more potent antiseizure effect during the metestrus and diestrus phases, indicated by elevated ITMS and ITTS and reduced seizure duration. With higher doses of resveratrol, MDA levels fell, whereas SOD and GPx levels rose. Furthermore, the pro-inflammatory cytokines TNF-α and IL-1β were diminished following resveratrol administration. The effect of resveratrol on GABA receptors varies with concentration and exhibits a biphasic nature; low concentrations improve receptor activity, whereas high concentrations suppress it (Sirotkin et al., 2019). Flavonoids show an agonistic effect on GABA receptors, and in the absence of GABA, they bind directly to the receptor, resulting in the opening of its channels; hence, it is noted that specific flavonoids have an active binding site on the GABA receptor.

The elevation of reactive oxygen species in the brain may lead to brain tissue oxidative damage which is accompanied by psychiatric and psychological issues (Tauffenberger and Magistretti, 2021). Although GABA receptors are important in the pathophysiology of epilepsy, oxidative damage is also increased in brain tissues in PTZ-induced seizures (Łukawski and Czuczwar, 2023). This PTZ-induced increase in oxidative stress is shown by changes in oxidative biomarkers. In both the entire brain and various brain regions (cortex, hippocampus) of PTZ-kindled mice and rats, there was an observed rise in LPO alongside a decline in GSH and antioxidant enzyme levels (SOD, CAT) (Cho et al., 2022).

Nonetheless, certain distinctions regarding antioxidative defense were noted between acute PTZ and PTZ kindling. Acute seizures of identical intensity do not produce the same changes. PTZ-kindled animals have diminished SOD and GPx activity and increased increase nitric oxide levels which impacts oxidative phosphorylation by blocking the mitochondrial respiratory during seizures (Kola et al., 2017). PTZ-induced subjects exhibited diminished discrimination skills, learning, and memory, alongside increased emotional stress and anxiety, which correlated with elevated hippocampal IL-1β and TNF-α levels (Wu et al., 2022). Resveratrol reduced liver inflammation mainly by causing a notable decline in the expression of several pro-inflammatory cytokines such as TNF-α, IL-1α, IL-1β, and IL-6. Resveratrol obstructs the transcription factor nuclear NF-κB, which initiates the inflammatory cascade. Resveratrol blocks the PI3K/Akt/mTOR pathway to trigger apoptosis. Moreover, it alleviates oxidative stress in liver tissue by significantly lowering MDA and nitric oxide levels while enhancing the concentrations of CAT, SOD, and reduced hepatic glutathione (Chupradit et al., 2022).

The molecular pathways underlying the antioxidant and anti-inflammatory effects of resveratrol may involve various signaling mechanisms. Recent research indicated that the ways in which resveratrol combats oxidative stress primarily involve the activation of the silent information regulator T1 (SIRT1) signaling pathway to limit the generation of reactive oxygen species (ROS), lower the expression of the receptor for advanced glycation end products (RAGE), activate adenosine monophosphate-activated protein kinase (AMPK), suppress the activation of nuclear factor kappa B (NF-κB), regulate the NF-E2-related-factor 2-Kelch-like ECH associated protein 1 (Nrf2-Keap1) signaling pathway, and reduce the expression of glutathione S-transferase (Deng et al., 2018).

Resveratrol therapy can boost SIRT1 deacetylase activity, thereby lowering the levels of acetylated-FoxO3a and reducing the oxidative stress induced by hyperglycemia in both in vitro and in vivo studies (Wang et al., 2017). Moreover, various studies have verified that Forkhead box O1 (FoxO1) is linked to oxidative stress as well. As MDA levels rose and SOD activity fell, the expression of FoxO1 decreased. However, following resveratrol treatment, FoxO1 expression increased, and the activity of SIRT1 deacetylase was boosted. These findings indicate that RSV may alleviate renal oxidative stress damage by influencing the SIRT1/FoxO1 signaling pathway (Deng et al., 2018). Resveratrol can markedly decrease the levels of IL-6, IL-1β, intercellular cell adhesion molecule-1 (ICAM-1), and various inflammatory cytokines in kidney tissue by modulating the AMPK/NF-κB, Akt/NF-κB, phosphoinositide 3-kinases/protein kinase B (PI3K/Akt) signaling pathways, along with the pancreatic insulin signaling pathways (Sadi et al., 2015). Moreover, resveratrol may suppress the expression of inflammatory cytokines like TNF-α and IL-6 by blocking the phosphorylation of extracellular signal-regulated kinase 1/2 (Hu et al., 2022)

Resveratrol has affinity to the benzodiazepine site on the GABA_A_ receptor which enhances the receptor sensitivity for endogenous GABA and preventing epileptic incidence (Ríos et al., 2022). The impacts of these two substances were examined on various GABA_A_ receptor subtypes present in *Xenopus laevis* oocytes through the use of the two-electrode voltage clamp method. These compounds exhibit varying activity patterns. For instance, when resveratrol is administered on its own, it generates a current of 22 nA in the α1β2γ2 L subtype of GABA_A_ receptor (which are linked to sedative and anticonvulsant properties) but does not influence the α5β3γ2 L subtypes (which are involved in learning and memory) and α2β2γ2 L (associated with anxiolytic and muscle relaxation effects); it also leads to a dose-dependent enhancement of the GABA-induced current (IGABA) in the α1β2γ2 L receptor (Ríos et al., 2022). During the acute phase, it is assumed resveratrol restored the silent and chronic phases of epilepsy, increased the expression of the hippocampal kainate glutamate receptor, and decreased the GABA_A-α1R_ levels (Li et al., 2017). Resveratrol therapy prevented the elevation of the glutamate/GABA ratio in the hippocampus caused by kainic acid (Li et al., 2017).

Scarce clinical trial information exists on phlorotannins or flavonoids pointing out a clear GABAergic implication. In a similar and old report Gupta et al. (Gupta et al., 2002) studied the protective effect of resveratrol against PTZ-induced seizures and based on their findings, resveratrol (40 and 80 mg/kg) has an antiepileptic role, and an adenosinergic mechanism may play a role in its anticonvulsant activity. Resveratrol (80 mg/kg) did not affect the duration of pentobarbitone-induced sleep, suggesting that the anticonvulsant effect of resveratrol is not due to its sedative properties (Gupta et al., 2002) and our findings support this report. Sedation is a significant side effect of many antiseizure medications, and the fact that resveratrol did not alter pentobarbitone sleeping duration suggests it can be used alongside antiepileptic medications without increasing sedation risks.

To the authors’ knowledge, there is no report on the antiseizure efficacy of resveratrol on PTZ-induced seizures during different phases of the estrous cycle. As mentioned, resveratrol positively influences estrogen metabolism and sex hormone-binding globulin in females (Briansó-Llort et al., 2022). Resveratrol acts as an analog of natural sex hormones and functions as an estrogen receptor agonist (Zaychenko et al., 2022). Estrogen and progesterone, essential hormones in the female reproductive system, are instrumental in the development of catamenial epilepsy. Variations in sex hormones during the sexual cycle can lead to a risk of seizures on certain days of the cycle. Menstrual epilepsy occurs more frequently during periods of increased estrogen levels or decreased progesterone levels (Mansour et al., 2021). The chances of menstrual epilepsy increase just before menstruation, probably due to lower progesterone levels, and during ovulation because of higher estrogen relative to progesterone levels. In a study on effects of resveratrol on receptor expression and serum levels of estrogen and progesterone, Han et al. (Han et al., 2021) reported resveratrol therapy lowered serum estradiol levels, elevated serum progesterone levels, improved estrogen receptor (ER) expression in the uterine stroma, diminished ESR_1_ gene expression, and boosted ESR_2_ gene expression and this effect is responsible for beneficial effect of the resveratrol in endometritis. Estradiol exhibits convulsant properties, whereas progesterone and its metabolites (predominantly allopregnanolone) serve an anticonvulsant function. As a result, hormonal fluctuations throughout the menstrual cycle may influence the frequency of seizures (Joshi and Kapur, 2019).

Resveratrol's anti-inflammatory properties are associated with the inhibition of NF-κB, which stops the release of proinflammatory cytokines and promotes inflammation by blocking downstream inflammatory mediators. Resveratrol affected mitogen-activated protein kinase (MAPK) signaling pathways and estrogen receptor-associated cascades to promote inflammation independently of NF-κB (Reda et al., 2022). A potential mechanism is via its antioxidant action, along with its inhibitory influence on p38 MAPKs, such as ERK and JNK. Further studies are required to comprehend how resveratrol affects inflammation. In conclusion, the results of this study indicate that resveratrol decreased seizures in PTZ-treated rats, though it was effective during the luteal phase compared to the follicular phase. The exact mechanism behind resveratrol's antiseizure effect remains unclear; however, its role in increasing progesterone levels and its antioxidants properties may contribute to this effect.

## CRediT authorship contribution statement

**Shahin Hassanpour:** Supervision, Project administration, Methodology. **Seyed Amirreza Dehghani:** Writing – original draft, Data curation. **Kiana Rastegar:** Writing – original draft. **Seyed Mehdi Sarmanrei:** Writing – original draft, Data curation.

## Ethics declaration

This study was conducted in accordance with the ARRIVE (Animal Research: Reporting of In Vivo Experiments) guidelines. The Islamic Azad University, Tehran, Iran granted an exemption. The authors provided the following explanation: study protocol was approved by Department of Veterinary Basic Sciences, SR.C., Islamic Azad University, Tehran, Iran

## Funding

None.

## Declaration of Competing Interest

No conflict of interest.

## Data Availability

Data is available by request
